# Mesenchymal Stem Cell-Derived Extracellular Vesicles and Their Therapeutic Potential

**DOI:** 10.1155/2020/8825771

**Published:** 2020-08-24

**Authors:** Ashley G. Zhao, Kiran Shah, Brett Cromer, Huseyin Sumer

**Affiliations:** ^1^Department of Chemistry and Biotechnology, Faculty of Science, Engineering and Technology, Swinburne University of Technology, John St., Hawthorn VIC 3122, Australia; ^2^Magellan Stem Cells P/L, 116-118 Thames St., Box Hill VIC 3129, Australia

## Abstract

Extracellular vesicles (EVs) are cell-derived membrane-bound nanoparticles, which act as shuttles, delivering a range of biomolecules to diverse target cells. They play an important role in maintenance of biophysiological homeostasis and cellular, physiological, and pathological processes. EVs have significant diagnostic and therapeutic potentials and have been studied both *in vitro* and *in vivo* in many fields. Mesenchymal stem cells (MSCs) are multipotent cells with many therapeutic applications and have also gained much attention as prolific producers of EVs. MSC-derived EVs are being explored as a therapeutic alternative to MSCs since they may have similar therapeutic effects but are cell-free. They have applications in regenerative medicine and tissue engineering and, most importantly, confer several advantages over cells such as lower immunogenicity, capacity to cross biological barriers, and less safety concerns. In this review, we introduce the biogenesis of EVs, including exosomes and microvesicles. We then turn more specifically to investigations of MSC-derived EVs. We highlight the great therapeutic potential of MSC-derived EVs and applications in regenerative medicine and tissue engineering.

## 1. Extracellular Vesicles

Extracellular vesicles (EVs) bearing nucleic acids, proteins, and lipids can be released into the extracellular space from eukaryotic cells, as well as from some prokaryotic cells [1]. These released EVs are lipid bilayer-bound nanoparticles and are found in many biological fluids such as serum, cerebrospinal fluid, saliva, urine, nasal secretions, and breast milk. They can also be collected in cell culture medium. Originally, EVs were regarded as cellular waste [2] but since have been shown to play important biological roles in cellular homeostasis and the spreading of biomolecules to neighbouring cells and tissues. Transported biomolecules can contribute to normal physiology or disease states or could be therapeutics to be delivered to damaged cells and tissues. For these reasons, EVs show significant potential in biotechnology [3–5]. Many different names have been used for extracellular vesicles, following several independent discoveries, which have led to confusing nomenclature. As the extracellular vesicle field has grown tremendously over the past few decades, the International Society for Extracellular Vesicles (ISEV) was launched in 2011, with the aim of advancing extracellular vesicle research globally. The term “extracellular vesicles” (EVs) was introduced by ISEV to describe preparations of vesicles isolated from biofluids and cell cultures [3]. Based on their size and biogenesis, EVs could be classified into three main subclasses: exosomes (40-120 nm), microvesicles (50-1000 nm), and apoptotic bodies (500-2000 nm) [6]. Both microvesicles and apoptotic bodies are directly shed from the plasma membrane but via different cellular processes, whereas exosomes are generated by the endocytic pathway and are originally considered to play a particularly important role in cell-to-cell communication [7].

## 2. Exosomes

**Figure 1:**
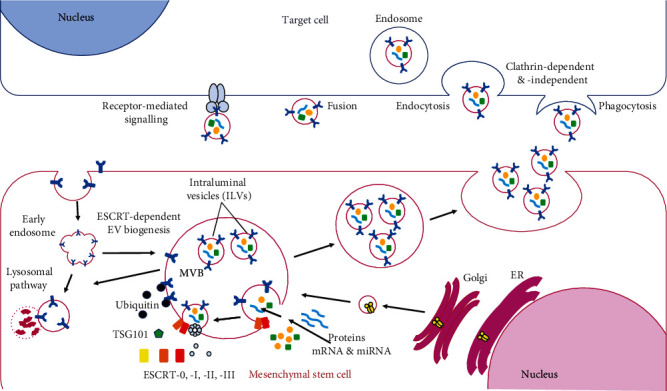
Extracellular vesicle biogenesis; ILVs invaginate from the outer endosomal membrane to bud into the lumen of endosomes through ESCRT-dependent/independent machineries during the maturation of MVB from the early endosome. Matured MVB is then transported to the cell periphery and fuses with the plasma membrane to release ILVs (exosomes). Exosomes together with microvesicles enter the target cells through signalling, fusion, and endocytosis pathways.

The term exosome was first used to describe membrane nanovesicles released from mammalian reticulocytes through the endosomal pathway in the 1980s [8–10]. Exosomes were originally thought to be waste products released by cells. In the subsequent decades, further research identified that exosomes have an important function as transport vehicles and can act to stimulate immune suppression of tumor growth [11, 12]. One of the important discoveries in the field was the presence of nucleic acids-mRNA and miRNA in exosomes and hence the ability to alter specific gene expression and protein translation in recipient cells [13]. Today, exosomes are recognised to play an important role in intercellular communication through transfer of proteins, lipids, and nucleic acids into recipient cells [6, 14, 15] (Figure 1).

### 2.1. Exosome Biogenesis

Many cellular processes are involved in the generation of exosomes. These include the production of microvesicular bodies (MVBs) and formation of intraluminal vesicles (ILVs) during early endosomal maturation into MVBs. This is followed by trafficking and fusion of MVBs with the plasma membrane, releasing ILVs extracellularly as exosomes [16]. Several cellular mechanisms are involved in the formation of ILVs and maturation of MVBs, including the Endosomal Sorting Complex Required for Transport (ESCRT) which involves both ESCRT-dependent and ESCRT-independent transport mechanisms, described below.

The best-described mechanism for the formation of ILVs is the ESCRT-dependent machinery [17, 18]. ILVs are formed from early endosomes by the inward budding of the limiting membrane and then scission of the narrow neck to release the bud into the endosomal lumen as a vesicle. ESCRT proteins sort ubiquitinated proteins into these buds [19]. The role of the four ESCRT complexes ESCRT-0, ESCRT-I, ESCRT-II, and ESCRT-III in the formation of ILVs in the interior of MVBs was well-described in the early 2000s [20–22]. The ESCRT-dependent mechanism starts from the interaction of the ESCRT-0 complex with ubiquitylated proteins, which are organized by clathrin into specialized endosomal subdomains [23]. Then, direct interaction between ESCRT-0 and TSG101 of the ESCRT-I complex recruits ESCRT-I and ESCRT-II and starts the inward budding of the ILVs into the lumen of the MVBs.

The ESCRT-I/ESCRT-II system is one core part of the ESCRT machinery, which functions as one branch of the ESCRT pathway to feed into ESCRT-III and the Vps4 scission machinery [19]. ESCRT-II recruits the ESCRT-III complex to develop a curved membrane-binding surface and line tubules extended away from the cytoplasm [24]. ESCRT-III also recruits the associated protein Alix for the recruitment of the deubiquitinating enzyme Doa4 [25]. Finally, ESCRT-associated proteins Vps4 and Vta1 cleave the ILV into free vesicles and disassemble ESCRT complexes [17]. Some ESCRT components and accessory proteins such as TSG101, HRS, and ALIX are retained in the ILVs and become important protein markers of exosomes. However, it is not clear whether they are specific markers for exosomes since ESCRT-I/II/III and their accessory molecules are associated with various other budding and membrane scission processes, such as microvesicle release, wound repair on the plasma membrane, neuron pruning, membrane abscission in cytokinesis, nucleus envelope reformation, and cellular autophagy processes [19]. Alternatively, ESCRT-0 has been specifically implicated in exosome secretion and is not yet described in plasma membrane budding and scission processes. Therefore, ESCRT-0 components might be more specific markers to demonstrate endosomal origin [26].

Interestingly, ILVs can still form in MVBs via ESCRT-independent mechanisms [27]. Many studies suggest that ESCRT-independent mechanisms are involved in ILV formation and exosome biogenesis. The ESCRT-independent mechanisms involve lipids (ceramide, cholesterol, and PLD2), tetraspanins, syntenins, or heat-shock proteins [23, 28–31]. For example, depleted ESCRT subunits such as Hrs, TSG101, Alix, or Vps4 and exosomes enriched in protein-lipid protein (PLP) and CD63 were still secreted through a ceramide-dependent sorting mechanism [15, 27]. Even though many studies have described significant contributions to ILV formation pathways, exosome biogenesis is still not exhaustively studied. Therefore, since current knowledge of exosome biogenesis is not fully specific to exosome secretion and is also not shown in all cell types [26], further studies on exosome biogenesis are still needed.

Once late endosomes become fully mature MVBs, they are transported to the cell periphery and fuse with the plasma membrane to release ILVs as exosomes [1, 32, 33]. The mechanisms of MVB mobilization, docking, and fusion involve a large network of proteins, including the actin cytoskeleton, microtubules, and associated molecular motors such as kinesins and myosins, molecular switches (small GTPases), tethering factors, and SNARE proteins [7, 32, 34–38]. Proteins and protein complexes organise the tethers and work together with Rab proteins to direct the vesicle targeting [34]. The activated Rab proteins (Rab GTPases) such as Rab7, Rab11, Rab27, and related Ral-1 regulate vesicle formation, trafficking, and fusion. They control movement through interaction of the vesicles with cytoskeletal components, tethering/docking these vesicles to the cell periphery [32, 37, 39–41].

MVB trafficking requires actin and microtubule cytoskeletons and motor proteins to transport and tether MVBs to the plasma membrane [33]. After docking of MVBs to the plasma membrane, soluble N-ethylmaleimide-sensitive factor attachment protein receptors (SNAREs) regulate the fusion of the MVB lipid bilayer with the plasma membrane to release ILVs [36]. SNAREs are the core fusion engine in membrane fusion and are recycled after each fusion event [35]. SNARE proteins are classified into four subfamilies based on their SNARE motifs; Qa-, Qb-, Qc-(t-), and R- (v-) SNAREs, which are highly conserved and diverged early in eukaryotic evolution [42]. They are assembled in a *trans* configuration and formed as helical core complexes, mediated by the SNARE motifs. The assembly starts at the N termini of the SNARE motif followed by a zipper-like fashion towards the C-terminal membrane anchors. The function of SNARE complexes is to provide the mechanical force exerted on the membrane to proceed with the fusion of two lipid bilayers and then distort membranes to form a fusion pore releasing ILVs of MVBs into the extracellular environment as exosomes [35].

## 3. Microvesicles

Similar to exosomes, many types of machinery are involved in microvesicle biogenesis. Unlike exosome biogenesis which has been intensively studied, microvesicle biogenesis has only recently started to emerge as a focus of study [43]. Microvesicles, also classified as ectosomes, are directly generated from the plasma membrane [44]. Microvesicles are generated by the formation of outward buds in specific sites of the membrane and then released into the extracellular space by fission [45]. Several molecular rearrangements are involved including changes in lipid and protein composition and even Ca^2+^ level at the specific sites of the membrane to elicit membrane budding [46, 47]. Ca^2+^ level changes alter the lipid composition of the plasma membrane, and the externalization of phosphatidylserine also plays a role in microvesicle formation [48].

Microvesicles have also been shown to be enriched in cholesterol and are raised from cholesterol-rich lipid rafts [49]. Furthermore, the depletion of cholesterol significantly reduces microvesicle shedding. Other factors such as molecular rearrangements in the plasma membrane, cell shape maintenance proteins, cytoskeletal elements, and their regulators are also involved in microvesicle biogenesis [50]. The regulators of actin dynamics, RhoA (a member of the small GTPases family), and its downstream-associated protein ROCK and LIM kinases are essential for microvesicle biogenesis [51]. A calcium-dependent enzyme, calpain, which regulates cytoskeletal proteins is involved in microvesicle shedding [52]. Inhibition of calpain could suppress PAK1/1 activation to decrease polymerization of actin, formation of filopodia, and furthermore interfere with the generation of microvesicles. ARF6 also plays a key role in microvesicle formation and shedding [53]. ARF6-GTP-dependent activation of phospholipase D recruits the extracellular signal-regulated kinase (ERK) to the plasma membrane, and then ERK phosphorylates and activates myosin light-chain kinase (MLCK) which is an important regulator of actin polymerization and myosin activity. This process is essential for microvesicle release, and inhibition of ARF6 could block microvesicle shedding. Both exosomes and microvesicles play important roles in physiological and pathological cellular processes.

## 4. EV Function

Endosomal exosomes were considered as the main mediators that affect recipient cells. However, it is difficult to efficiently separate exosomes from other subtypes of EVs by current isolation methods, so it is difficult to definitively assign a function to a particular type of vesicle. Furthermore, not only do the formation and secretion of ILVs employ multiple mechanisms, resulting in heterogeneous exosomes, but other EVs also overlap in their biophysical properties [54]. Moreover, there is currently no consensus on markers to distinguish exosomes from other EVs.

The techniques used to isolate small EVs result in a heterogeneous mix of sizes, origin, and molecular composition, with an unknown portion of them being exosomes [55]. Therefore, they may contain a mixture of endosomal and nonendosomal small EVs [56] and even some nonvesicular molecules such as various dense lipoproteins [57]. Nevertheless, many studies have discovered a significant function of EVs to target cells and demonstrated their potential in many pathophysiological fields such as cancer, immune responses, various diseases, and regenerative therapeutics [5, 6]. Even though there are many studies that describe the function of exosomes, most of these studies may contain a mixture of EVs with different subtypes due to their preparation method, so the observed function, assigned to exosomes, may be elicited by multiple EV types [26].

EVs carry proteins, lipids, and nucleic acids and can be released by most cells and taken up by recipient cells to trigger various phenotypic effects [58]. The lipid bilayer of EVs can protect their content, transit through the extracellular fluid, and internalise into recipient cells. Different recipient cell types take up heterogeneous EVs through different pathways which are highly specialised and specific processes [59]. EVs bind to appropriate receptors on target cells through receptor-ligand interaction and enter these cells through three major EV uptake pathways: signalling, fusion, and endocytosis [43, 59].

Many studies have shown the diverse biological functions of EVs. EVs released by B lymphocytes present MHC-peptide complexes to specific T cells which suggested that EVs played a role in adaptive immune responses [60, 61]. Proteins and mRNAs of EVs can be transferred into target cells, and mRNAs can be translated into corresponding proteins [62]. For example, selective mRNAs and miRNAs were found in mast cell EVs and involved in the immune response [13].

Genetic communication between cells might also occur via the trafficking of EVs through the systemic circulation, similar to how hormones impact their recipient cells. EVs derived from stem cells play a pivotal role in tissue regeneration [63, 64]. EVs not only play important roles in many aspects of biology such as intercellular vesicle traffic, immunity, neurobiology, and microbiology but also have important roles in disease pathogenesis such as tumor progression, neurodegenerative propagation, and HIV and prion spread [6, 65]. For example, tumor cells can release EVs into the microenvironment to elicit tumor progression via numerous mechanisms such as promoting angiogenesis, suppressing immune responses, and tumor cell migration in metastases [65, 66]. More recently, mesenchymal stem cells have been shown to be prolific producers of EVs and have been investigated for their potential therapeutic applications.

## 5. Mesenchymal Stem Cells and EVs

Mesenchymal stem cells (MSCs) are multipotent stem cells derived from mesenchyme, which develops from the mesoderm [67]. MSCs are capable of self-renewal and differentiation into skeletal and connective tissues such as the bone, fat, cartilage, and muscle [68]. The main roles of resident MSCs in adults are self-repair and to maintain cellular tissue homeostasis. Due to their plastic adherence properties when cultured *in vitro*, MSCs can be easily isolated from various organs and tissues such as the bone marrow, adipose tissue, muscle tissue, skin, teeth, periosteum, trabecular bone, synovium, skeletal tissues, brain, spleen, liver, kidney, thymus, pancreas, and blood vessels [68, 69]. MSCs are considered to be ideal candidates for tissue regeneration and tissue engineering, and interest in their biological roles and clinical potential has dramatically increased over the last three decades [70].

There are over two thousand clinical trials registered on ClinicalTrials.gov investigating therapeutic applications of MSCs in many diseases, such as bronchopulmonary dysplasia, multiple sclerosis, autoimmune diseases, Alzheimer's disease, liver diseases, osteoarthritis, kidney disease, myocardial infarction, and graft versus host disease. Initially, the therapeutic applications of MSCs were investigated to replace injured cells, based on their differentiation potential. However, less than 1% of the transplanted MSCs could reach the target tissue, such as the infarcted myocardium in treatment of myocardial infarction [71]. Nonetheless, MSCs restored heart function more rapidly compared to the slow and inefficient differentiation process of cardiomyocytes [72]. MSCs have also been shown to be effective in treating degenerative diseases such as osteoarthritis for both animals and humans [73, 74]. Furthermore, it has been demonstrated that MSCs can be effective in the modulation of immune responses, anti-inflammatory affect, tissue repair, and regeneration in many therapeutic applications *in vitro* and *in vivo*. Therefore, MSCs are proposed to exert their beneficial effects by paracrine secretion rather than from their differentiation [75, 76], for which most MSC clinical trials were rationalized. However, to date, none of the identified soluble secreted mediators alone are able to sufficiently mediate the MSC therapeutic effects [77]. Subsequently, many studies have shown that the paracrine effects of MSCs were mediated in part by the secretion of EVs [63, 78]. Thus, extracellular vesicles derived from MSCs might be a safer cell-free alternative to cell therapy [79]. More recently, the research focus on the mechanism of therapeutic action of MSCs, which was previously attributed to their differentiation and paracrine efficacy, has now focused on the role of EVs. MSC-derived EVs play an important role in the regulation of normal physiological, tissue regenerative, and pathological propagation processes, and MSCs are considered to be prolific producers of EVs when compared to other cell types [80].

MSC-derived EVs have been shown to contain at least 730 different proteins [81]. These proteins reflected both features of MSCs and EVs. For example, 53 proteins of MSC-derived EVs were related to self-renewal genes associated with MSCs, and 25 proteins were differentiation genes of MSCs. In their study, Kim et al. (2014) showed that MSC-derived EV proteins included not only surface markers of MSCs but also MSC-specific proteins involved in signalling pathways to facilitate self-renewal and differentiation. MSC-derived EVs also contain proteins associated with EV biogenesis, trafficking, docking, and fusion. Furthermore, EV proteins such as the surface receptor PDGFRB, EGFR, and PLAUR; signalling molecules of RAS-MAPK, RHO, and CDC42 pathways; cell adhesion molecules; and additional MSC antigens are associated with promotion and modulation of MSC therapeutic potential. These proteins may play a role in the efficacy of MSC-derived EVs in tissue repair and tissue regeneration. Even though EV miRNAs were estimated to be less than one copy per EV [82], some EVs might be enriched with certain miRNAs. 171 miRNAs were identified in MSC-derived EVs [83]. The most abundant 23 miRNAs could target 5481 genes to regulate many specific pathways and biological processes, such as miR-130a-3p and miR-199a, which induce cellular proliferation, promote angiogenesis, and inhibit apoptosis. Furthermore, the proteome of purified MSC exosomes as profiled by mass spectrometry and antibody arrays contains 938 unique gene products found in the exosome database website http://exocarta.org that encompass a wide range of biochemical and cellular processes including cellular communication, structure and mechanics, inflammation, exosome biogenesis, tissue repair and regeneration, and metabolism [84].

## 6. Therapeutic Applications of Mesenchymal Stem Cell-Derived Extracellular Vesicles

To date, the therapeutic potential of MSC-derived EVs has been studied in both animal models and various clinical applications for many disease areas, such as cardiovascular disease, acute kidney injury, liver disease, lung disease, cutaneous wound healing, and cancer suppression [72, 85–87]. EVs have also been tested as potential diagnostic tools, antitumor therapeutics, drug delivery vehicles, and vaccines [85, 88]. Here, we focus on the therapeutic potential of MSC-derived EVs in a number of applications in regenerative medicine.

One of the first reports of MSC-derived EVs was of those derived from human bone marrow MSCs. These EVs had a beneficial impact on tubular epithelial cells through delivering mRNA cargo to activate regenerative programmes and resulted in recovery from acute kidney injury *in vitro* and *in vivo* [89]. Furthermore, intravenous administration of human MSC-derived EVs had the same efficacy as MSCs themselves on the treatment of acute kidney injury by inhibiting apoptosis and stimulating tubular cell proliferation in a rat model [86]. They also protected the kidney from the development of chronic injury, which highlights the potential of MSC-derived EVs for regenerative medicine.

Recent studies include the use of MSC-derived EVs for the treatment of a number of neuropathological diseases, such as multiples sclerosis [90] and Alzheimer's disease [91]. In a mouse model of multiple sclerosis, the mice were treated with saline, placenta MSCs, and low-dose (1.0 × 10^7^) or high-dose (1.0 × 10^10^) human placenta MSC-derived EVs. [90]. Both MSCs and MSC-derived EVs showed regenerative effects and prevented oligodendroglia degradation and demyelination, resulting in motor function improvement. Importantly, animals treated with high-dose MSC-derived EVs or MSCs showed similar clinical outcomes, demonstrating that MSC-derived EVs possess the same therapeutic potential as MSCs. Another preclinical study showed that MSC-derived EVs could be a therapeutic strategy for the treatment of currently incurable Alzheimer's disease [91]. After 28 days of injection of 10 *μ*g EVs and 1 × 10^6^ MSCs separately into two groups of mice with induced Alzheimer's disease, both groups had similar beneficial effects in improvement of neurogenesis and cognitive function.

MSC-derived EVs are capable of reducing the infarct size of myocardial injury through modulating the injured tissue environment, inducing angiogenesis, promoting proliferation, and preventing apoptosis [63]. The therapeutic effects of MSC-derived EVs on myocardial infarction have been demonstrated in a mouse model [92]. MSC-derived EVs could reduce infarct size to preserve cardiac function for an extended period through rapid activation of multiple cardioprotective pathways.

The function of MSC EVs in cartilage repair has been studied by investigation of the effects of human MSC-derived EVs on chondrocyte survival *in vitro* [93]. The chondrocytes could quickly endocytose the labelled MSC-derived EVs and rapidly phosphorylate AKT and ERK in chondrocytes within 1 hour to elicit the cellular proliferation of chondrocytes. MSC-derived EVs enhanced regeneration of the damaged cartilage through inducing proliferation, migration, and matrix synthesis of chondrocytes, attenuating apoptosis and modulating immune reactivity. Furthermore, intra-articular injection of 100 *μ*g/100 *μ*l of embryonic MSC-derived EVs could efficiently repair osteochondral defects in a rat model [94]. The results from the MSC-derived EV treatment group showed hyaline cartilage regeneration by the end of 12 weeks. In contrast, the defects of controls treated with PBS were filled with fibrous and noncartilaginous tissue. Additionally, there were no adverse inflammatory responses in this experiment. In a preclinical study, the efficacy of MSC-derived EVs secreted from synovial membrane was compared to induced MSC-derived EVs in the treatment of mouse osteoarthritis (OA) [95]. Intra-articular injection of only 8 *μ*l of EVs (1.0 × 10^10^/ml), from either source, into collagenase-induced OA mice attenuated OA. MSC-derived EVs showed a more significant effect than synovial membrane MSC-derived EVs. Furthermore, EVs from adipose tissue-derived MSCs could repair damaged cartilage through increasing the proliferation and migration of chondrocytes in a rat model of OA [96]. These numerous studies demonstrate the possibility of treating chronic conditions with MSC-derived EVs to address current unmet medical needs.

### 6.1. Alternate Therapeutic Delivery Methods of MSC-Derived EVs

As researchers have begun to unlock the therapeutic potential of MSC-derived EVs in the field of regenerative medicine, alternate delivery methods are being explored. These include the encapsulation of EVs in hydrogels or incorporation into biodegradable scaffolds such as polylactide (PLA) and polyethyleneimine (PEI). These methodologies represent ways of cell-free delivery methods with the benefits of MSCs, which can be sustained over long periods of time.

Hydrogels are a 3D network of polymers with hydrophilic properties that can swell in an aqueous solution and absorb biologic fluids and therefore have the potential to act as delivery vectors in tissue engineering. A biodegradable hydrogel was used to encapsulate ES cell-differentiated MSC-derived EVs in a rat hepatic regeneration model [97]. The EVs were encapsulated in PEG hydrogels, which acted as a sustained-release EV depot to treat liver disease in rats [97]. The MSC-derived EV-laden hydrogels could gradually release EVs and result in accumulation in the liver for one month, compared to 24-hour clearance after conventional bolus injection. This study not only demonstrated the antiapoptosis, antifibrosis, and regenerative properties of MSC-derived EVs but also demonstrated a sustained systemic delivery method which could be employed for treatment of a variety of diseases.

Alternatively, EVs can be incorporated into solid 3D scaffolds when modelling structures such as bone. In a rat model of calvaria bone tissue damage, MSC-derived EVs were delivered on 3D PLA and PEI scaffolds to determine their ability to repair bone lesions [98]. Human MSCs, MSC-derived EVs, and 3D PLA or PEI-engineered EVs were evaluated in a number of combinations for their capability for bone defect regeneration *in vitro* and *in vivo*. It was found that there was more host tissue in-growth in the implant of 3D-PLA+MSC EV and 3D-PLA+EV+MSC samples than 3D-printed PLA scaffolds only and 3D-PLA+MSC samples. Abundant ECM, formation of nodules, and visible blood vessels in 3D-PLA+MSC EV, 3D-PLA+EV+MSC, 3D-PLA+PEI-EV, and 3D-PLA+PEI-EV+MSC samples were reported. This finding demonstrates that MSC-derived EVs could contribute to osteogenic regeneration, improve the mineralization process, and develop an extensive vascular network. Furthermore, the calvarial bone defect was completely repaired in 3D-PLA+EV+MSC, 3D-PLA+PEI-EV, and 3D-PLA+PEI-EV+MSC samples when evaluated for up to 16 weeks, which demonstrates the potential of MSC-derived tissue engineering for the treatment of bone defects. In another study on cartilage regeneration, MSC-derived EVs were evaluated using 3D-printed ECM and Gelatin-Methacryloyl (GelMA) hydrogels in a rabbit OA model [99]. The 3D-printed ECM/GelMA/EV scaffold had the best therapeutic effect in cartilage regeneration when compared to 3D-printed GelMA and 3D-printed ECM/GelMA scaffolds. The defect region with the 3D-printed radially oriented ECM/GelMA/EVs had facilitated cartilage regeneration and repaired tissue with a mixture of fibrocartilage and hyaline-like cartilage. These studies suggest a promising application of MSC-derived EVs in 3D printing for tissue engineering of bone and cartilage.

### 6.2. Clinical Trials Using MSC-Derived EVs

**Figure 2:**
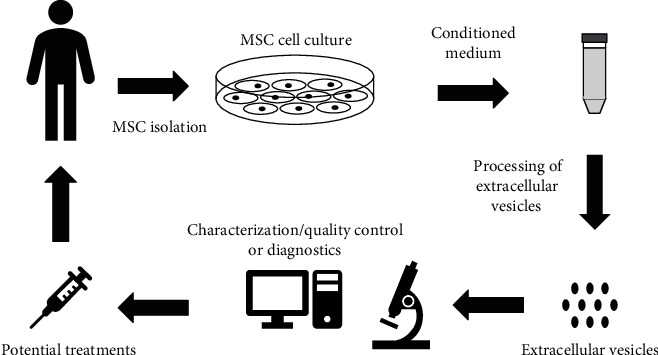
Workflow of MSC-derived EVs for therapeutic and diagnostic applications. MSCs can be isolated from patients from a variety of tissues. MSCs are cultured *in vitro*, and the conditioned culture medium is collected and subjected to extracellular vesicle isolation and/or purification. The isolated MSC-derived EVs can be used for diagnostic purposes or undergo quality control before being used in autologous and/or allogeneic therapeutics.

Overall, MSC-derived EVs have been evaluated for their therapeutic potential for the treatment of various diseases both *in vitro* and in animal models. Based on these results and findings, a number of clinical trials have begun to evaluate the therapeutic potential of MSC-derived EVs for the treatment of particular diseases and the procedure similar as in Figure 2. Using the key search words of “exosomes” and “extracellular vesicles” in the clinical trials website (https://clinicaltrials.gov/) reveals 172 and 51 registered clinical trials, respectively. Although some of these studies include MSC-derived EVs, very few clinical studies have been published. MSC-derived EVs have improved therapy-refractory graft-versus-host disease (GvHD) in patients [80]. These MSC-derived EVs were isolated from allogeneic MSC-cultured medium and delivered to steroid-refractory GvHD patients in escalating doses. The clinical GvHD symptoms significantly declined shortly after the start of MSC-derived EV treatment. The GvHD patients were stable and had no side effects. Another clinical trial displayed efficacy outcomes using EVs derived from umbilical cord MSCs to treat chronic kidney disease [100]. These results demonstrated that MSC-derived EVs could safely improve the inflammatory immune reaction and overall kidney function in chronic kidney disease patients through MSC EV administration in two doses, the first intravenous and the second intra-arterial.

Based on the preclinical and clinical studies, human MSC-derived EVs are considered as promising products in regenerative medicine and tissue engineering. Many studies have compared the beneficial effects of MSCs and MSC-derived EVs and showed that they had similar therapeutic outcomes. This indicates that MSC-derived EVs possess the same therapeutic potential as MSCs. The use of MSC-derived EVs might serve as an alternative, cell-free therapy over MSC transplantation for tissue regeneration [81] and have “off-the-shelf” therapeutic potential. Furthermore, clinical applications of MSC-derived EVs are advantageous over MSC cell-based therapy, as they have lower immunogenicity, capacity to cross biological barriers, and less safety concerns, such as the possibility of MSC differentiation or tumor generation [88, 101, 102]. The preclinical results using MSC EVs in tissue engineering have given exciting promise to their use as powerful tools as therapies to tackle a wide a range of unmet disease burden.

Despite the progress in the field, the EV isolation method may yield different EV subtypes as they coexist but may differ in their functional properties [103]. The heterogeneity of MSCs which include tri-, bi, and unipotent populations [104] needs to be addressed as they may impact on therapeutic outcomes of trials using EVs derived from different MSC populations. Not only are EVs highly heterogeneous, but they also have shown to result in various outcomes; stressed MSCs cultured in serum-deprived media secrete tumor-supportive EVs [105], and therefore, some caution is advised when using MSC-derived EVs for regenerative applications and more research is required. It should also be noted that some of the clinical studies have been terminated without publication. Furthermore, some experiments have demonstrated better results when using MSCs and MSC-derived EVs together, compared to the cells or EVs alone [90, 98]. Other considerations include the dose requirement, as some studies required higher doses of EVs or multidose injections to achieve significant therapeutic outcome [90, 100]. Another shortcoming is the half-life of EVs. Cellular therapies using MSCs are able to continuously release the beneficial paracrine factors (including EVs), while EVs have a relatively short half-life and therefore might be unable to retain sufficient levels present at the defect region [103]. However, this drawback might be offset by using alternate delivery methods such as bioengineered scaffolds, such as PEI, encapsulation with PEG hydrogels, or GelMA to maintain the sustained release of the MSC-derived EVs [97–99]. These bioengineering techniques for EV delivery might open up new avenues for therapeutic application.

Along with rapid development of the EV field, MSC-derived EVs have gained significant attention for their use in regenerative medicine. MSC-derived EVs bearing proteins, lipids, and RNAs could impact the target cells to exert their therapeutic effects. The cellular fate of EVs is still not well understood [26], and many questions of MSC-derived EV biodistribution are unanswered. Furthermore, the therapeutic mechanism of MSC-derived EVs still remains elusive [106]. Many MSC-derived EV studies *in vitro* and *in vivo* have verified that they are capable of enhancing tissue repair and mediating regeneration in various diseases and enhancing therapeutic outcomes. MSC-derived EVs have the theoretical advantages of being a safer regenerative tool when compared to cell-based therapies. However, we are in the early stage of using MSC-derived EVs in regenerative medicine. Standardised techniques for culture conditions and large-scale culturing, effective isolation, optimal dosing, and safe storage need to be methodically determined before large-scale clinical applications. We believe that MSC-derived EVs hold great promise in cell-free therapy, with the potential to be applied in a wide range of diseases.
